# Genomic Sequencing and Biological Characteristics of a Novel *Escherichia Coli* Bacteriophage 9g, a Putative Representative of a New Siphoviridae Genus

**DOI:** 10.3390/v6125077

**Published:** 2014-12-19

**Authors:** Eugene E. Kulikov, Alla K. Golomidova, Maria A. Letarova, Elena S. Kostryukova, Alexandr S. Zelenin, Nikolai S. Prokhorov, Andrey V. Letarov

**Affiliations:** 1 Laboratory of microbial viruses, Winogradsky Institute of Microbiology, Russian Academy of Sciences, prosp. 60-letiya Oktyabrya, 7/2, 117312 Moscow, Russia; E-Mails: eumenius@gmail.com (E.E.K.); alusik81@mail.ru (A.K.G.); maria.levina@gmail.com (M.A.L.); prokhoroff@gmail.com (N.S.P.); 2 SRI of Physical-Chemical Medicine, Russian Federal Medical and Biological Agency, ul. Malaya Pirogovskaya, 1a, 119435 Moscow, Russia; E-Mails: el-es@yandex.ru (E.S.K.); zeleniy.spb@gmail.com (A.S.Z.); 3 Kazan Federal University, ul. Kremlevskaya, 18, 420008 Kazan, Russia

**Keywords:** bacteriophage genome, modified DNA base, pseudolysogeny, horse, feces

## Abstract

Bacteriophage 9g was isolated from horse feces using *Escherichia coli* C600 as a host strain. Phage 9g has a slightly elongated capsid 62 × 76 nm in diameter and a non-contractile tail about 185 nm long. The complete genome sequence of this bacteriophage consists of 56,703 bp encoding 70 predicted open reading frames. The closest relative of phage 9g is phage PhiJL001 infecting marine alpha-proteobacterium associated with *Ircinia strobilina* sponge, sharing with phage 9g 51% of amino acid identity in the main capsid protein sequence. The DNA of 9g is resistant to most restriction endonucleases tested, indicating the presence of hypermodified bases. The gene cluster encoding a biosynthesis pathway similar to biosynthesis of the unusual nucleoside queuosine was detected in the phage 9g genome. The genomic map organization is somewhat similar to the typical temperate phage gene layout but no integrase gene was detected. Phage 9g efficiently forms stable associations with its host that continues to produce the phage over multiple passages, but the phage can be easily eliminated *via* viricide treatment indicating that no true lysogens are formed. Since the sequence, genomic organization and biological properties of bacteriophage 9g are clearly distinct from other known Enterobacteriaceae phages, we propose to consider it as the representative of a novel genus of the Siphoviridae family.

## 1. Introduction

The horse gut ecosystem was found to be the habitat of a large spectrum of different virulent bacteriophages, though [1,2,3] while the prevalence of temperate phages appears to be low in the free phage pool of horse feces. Our preliminary data [1,4] suggests that the ecosystem of the horse gut may be more open for acquisition of the new bacteriophage types than the intestinal microbiomes of humans and some other mammal species where the associated viromes instead are remarkably stable [5,6,7]. The unusually high strain-level diversity of coliform bacteria in the individual gut microbiomes in horses [8] is also suggestive of an active lateral flow of bacterial strains between adult animals. Thus, the horse gut may act as a natural enrichment cultivator that facilitates the detection and identification of the novel types of bacterial viruses, especially the phages of family Enterobacteriaceae.

The current International committee on taxonomy of viruses (ICTV) classification of bacteriophages [9] establishes some phage genera on the basis of morphology, conserved genomic synteny, and homology of amino acid sequences of phage-encoded proteins. There is a tendency to split larger genera that include very distantly related phages and to introduce subfamily levels into phage taxonomy. For example T-even related bacteriophages are currently split into at least two genus-level taxons: T4-like viruses and Schizo-T4-like viruses that do not include more distant T4-relatives such as “exo-T-evens” represented by marine cyanophages [10]. In the Siphoviridae family, subfamilies are not yet established, although some Siphoviridae genera include phages that are more distant from each other than, for example, “T-even” from “Schizo-T-even” [11] (see also [9]). One can thus assume that these genera will be split upon the identification of novel representatives of different clusters found within them.

The Yualikeviruses genus currently includes three species: *Pseudomonas* phage YuA, *Pseudomonas* phage M6 and phage phiJL001. [11,12]. Here we report the genome sequence and biological characterization of a novel coliphage 9g. Coliphage 9g shares weak homology with some of the proteins of bacteriophage phiJL001, but nonetheless infects a phylogenetically distant host. Being clearly distinct from other Enterobacteriacea phages by its genomic sequence and properties, phage 9g may be considered as representative of a novel genus-level taxon distantly linked to the YuA-like phages of Alpha-proteobacteria.

## 2. Materials and Methods

### 2.1. Phage and Bacterial Strains and Their Cultivation

*E. coli* C600 and *E. coli* 4sAs strains were from our collection. *E. coli* 4sAs is a derivative of the field isolate *E. coli* 4s [2] isolated from a pseudolysogenic association started with the phage G7C and *E. coli* 4s [12]. This strain has O-polysaccharide chain reduced to a single unit (our unpublished data). Bacteriophage T4 stock used for electron microscope magnification control was from our laboratory collection.

All the strains were propagated on Luria-Bertrani (LB) medium containing per L: Tripton (Amresco, Solon, OH, USA), 10 g; Yeast extract (Amresco), 5g; and NaCl, 5g. This medium was supplemented by 15 g per L of bacto-agar for plates or with 6 g of the bacto-agar per L for the soft agar used in double-layer plates for bacteriophage cultivation or titration [13].

To produce high titer phage stocks of *E. coli* C600, 60 ml culture was grown in LB in 250 ml flask at 37 °C with agitation at 250 rpm. When the culture reached OD_600_ of *ca.* 0.6 it was inoculated with 10^7^ PFU of phage per ml and the incubation was continued until visible lysis occurred (or overnight). Chloroform was then added and cell debris precipitated by centrifugation at 15,000 *g* for 15 min. Typical titer of the phage stock was about 10^11^ PFU.mL^−1^.

### 2.2. Bacteriophage Isolation

Isolation of bacteriophage 9g from horse feces was performed following the procedure described earlier [1]. The phage was purified via repeated plaque re-isolation.

### 2.3. Transmission Electron Microscopy (TEM)

TEM study of the phage specimen was done in accordance with general guidelines given in. [14,15]. We used copper EM grids (400 mesh, JEOL Inc., Tokyo, Japan) coated with collodion film (made on deionized water surface from a drop of 2% trinitrocellulose (Serva, Heidelberg, Germany) solution in anhydrous amyl acetate (Reachim, Moscow, Russia) and strengthened with a vacuum-deposited carbon layer. By our observations, collodion membranes are preferable over Formvar™ (Monsanto Chemical Company, St. Louis, MO, USA) films due to enhanced phage adsorption and higher mechanical strength. A fresh phage lysate with a titer about 10^10^ PFU.mL^−1^ was adsorbed to a coated grid for 15 min, taken off with a piece of filter paper, washed briefly in deionized water and stained with 2% filtered aqueous solution of uranyl acetate (Serva, Heidelberg, Germany). The stained grids were observed in JEOL 100CX transmission electron microscope (acceleration voltage 80 kV), and the results were photographed on sheet film (AGFA Gevaert, Mortsel, Belgium).

### 2.4. Adsorption Curve Determination and Single Burst Experiment

To measure phage adsorption kinetics, liquid culture of *E. coli* C600 was grown to an OD_600_ of about 0.2. The culture was diluted and plated to determine the CFU count. Then the appropriate dilution of phage stock was added to obtain a phage concentration about 5 × 10^5^ PFU.mL^−1^. Aliquots of 10 μL were collected at different time points and were immediately diluted 1:100 into physiological saline to slow down adsorption. Diluted samples were kept on ice until the end of the experiment and then were centrifuged for 1 min using a table-top microcentrifuge at 12,000 *g*. Aliquots of 50 or 200 μL of supernatant were plated using double layer technique to determine the concentration of free phage particles. The sample “0” collected immediately after mixing of the added phage was plated without centrifugation to enumerate the total concentration of phage added. The entire experiment was performed in triplicate.

### 2.5. Generation and Analysis of Phage-Host Pseudolysogenic Associations (PA)

To start the PAs, material from phage 9g plaques was transferred using toothpicks to fresh LB plates. After overnight incubation, growth of the bacterial culture was observed. The cultures were maintained in multiple passages on solid LB medium.

Testing for phage production was performed by toothpick transfer onto fresh lawns of appropriate host strains. Alternatively, suspensions of the PA cultures were prepared in LB broth and spotted over the lawn of the test strain.

The viricide treatment was used to separate the PA bacteria from phages. The viricide buffer was a strong tea infusion prepared as described in [17], using the action of tea tannins against phage proteins. In brief, the procedure was as follows: 50 g of black tea leaves were added to 100 mL of boiling Milli-Q deionized water and incubated at 100 °C for 20 min. The extract was then cooled to room temperature and filtered through a 0.45 membrane (Millipore, Billerica, MA, USA). The sterile extract was stored at 4 °C (the extract was heated to 40 °C before use when a precipitate had formed and then cooled to room temperature). The activity against free bacteriophage 9g particles was controlled each time the extract was used. The incubation of the phage stock containing 10^10^ PFU.mL^−1^ with 1:3 of the final volume of the viricide extract resulted in the drop of the viable phage titer to 10^3^ PFU.mL^−1^ after 5 min incubation at room temperature and to below 10^1^ PFU·mL^−1^ after 10 min.

### 2.6. Phage DNA Extraction and Sequencing

High titer lysates (>10^10^ PFU.mL^−1^) were incubated for 1 h at 37 °C with 0.001 volume of nuclease mix (2000 U DNAse I and 100 mg.mL^-1^ pancreatic RNAse, 100 mM MgCl_2_ in 50% glycerol), cleared by high-speed centrifugation (Beckman-Coulter, Brea, CA, USA) JA-20 rotor, 25,000 *g* for 30 min, and pelleted from supernatants in an ultracentrifuge (Beckman SW-28 rotor) at 100,000 *g* for 1 h. The homogeneity of the sample was assessed by transmission electron microscopy. The DNA was extracted from the precipitated phage by phenol extraction [16]. The phage genomic DNA library were prepared and sequenced using Ion Torrent PGM (Life Technology, Waltham, MA, USA) genetic analyzer and the kits Ion Xpress™ Plus Fragment Library Kit (Life Technology), Ion Xpress™ Barcode Adaptors 1-16 Kit, Ion PGM™ Template OT2 400 Kit, Ion PGM™ Sequencing 400 Kit, Ion 318 Chip Kit V2 according to the manufacturer’s instructions. The medium length of the individual reads was 290 bp, the average coverage of the bacteriophage 9g genome was 111. The genome was assembled *de novo* by use of CLC GW 6.0 software (CLC Bio, Aarhus, Denmark). Potential open reading frames (ORFs) were identified by use of GeneMarkHMM [17,18], DNAStar (DNAStar, Inc., Madison, WI, USA) and BASYS software packages [19,20] and subsequently analyzed manually. Putative functions of the ORFs were predicted using BLAST (NCBI) [21,22] and HHPred [23], ARAGORN [24,25] and tRNAscan-SE [26,27] resources were used to search for tRNA genes.

The genome sequence of Enterobacteria phage 9g is available under GenBank accession number NC_024146.1.

## 3. Results

### 3.1. Bacteriophage 9g Isolation and Its Host Range

Bacteriophage 9g was isolated from a horse feces sample using the *Escherichia coli* K12 derivative C600 [28]. It was present as a contamination in a plaque formed by a T5-like virus. Bacteriophage 9g was able to infect the laboratory *E. coli* strains JM109, BL21, and Be/1. We also were able to propagate it on the derivatives of the *E. coli* 4s strain selected for the resistance to bacteriophage G7C [4]. The later phage-host system was isolated from the feces collected from the same animal that served as a source of bacteriophage 9g isolate [1]. G7C bacteriophage infection pressure frequently selects for various mutants defective for O-antigen synthesis [29]. These mutants were susceptible to bacteriophage 9g while *E. coli* 4s wild type was resistant. It should be mentioned that all currently known hosts for bacteriophage 9g are O-antigen deficient mutants. Several environmental *E. coli* isolates tested did not support the 9g plaque formation (Golomidova, unpublished results). It is likely that some O-antigen type strains can be recognized by the side tail fibers of this virus, but these have not yet been identified.

### 3.2. Virion Morphology

**Figure 1:**
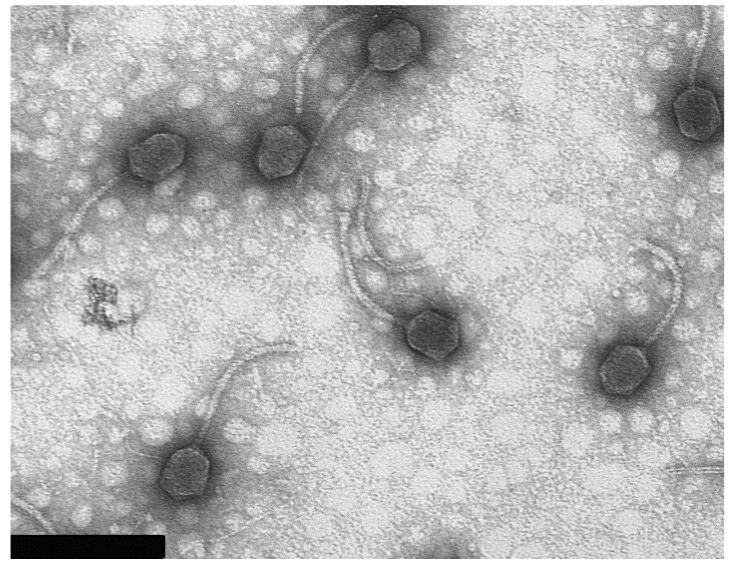
Bacteriophage 9g particle morphology. The bacteriophage T4 particles were used for calibration of the instrument magnification (the length of T4 tail is 113 nm [24]). Bar represents 200 nm.

Electron microscopy revealed that bacteriophage 9g belongs to the Siphoviridae family. It has a slightly elongated head of *ca.* 62 × 76 nm and 185 nm long non-contractile tail (Figure 1). The values are averaged out of 20 particles measured.

### 3.3. Life Cycle Parameters

**Figure 2:**
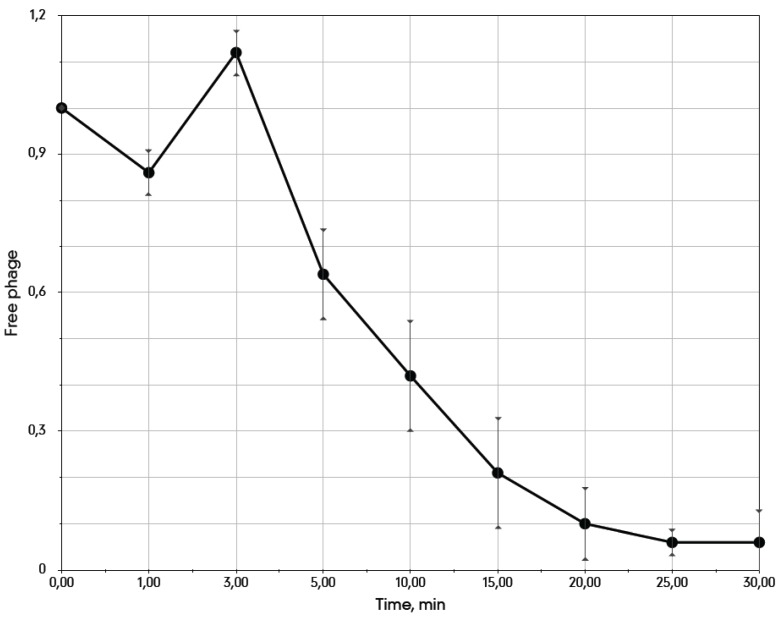
The adsorption curve of the bacteriophage 9g on *E. coli* C600 cells at 37 °C. The average cells density is 3.1 × 10^7^ CFU·mL^−1^.

We determined the bacteriophage 9g adsorption curve on *E. coli* C600 cells at 37°C (Figure 2). The phage interacts with cells at a moderate rate (adsorption constant of 5.3 × 10^−9^ mL.min^−1^). An unusual phenomenon of apparent detachment of the fraction of the initially adsorbed phage was repeatedly observed in a 0–5 min frame. The free phage titer at 3 min (Figure 2) was even slightly higher than that at the 0 time point that may reflect a disaggregation of the phage particles resulting from the initial interaction with the host cells. We currently do not have a coherent explanation for such kinetics. As all of the hosts currently known for 9g are O-antigen deficient mutants, it is possible that the adsorption rate on the appropriate O-antigen producing cells could be higher due to recognition by the phage side tail fibers [30,31].

**Figure 3:**
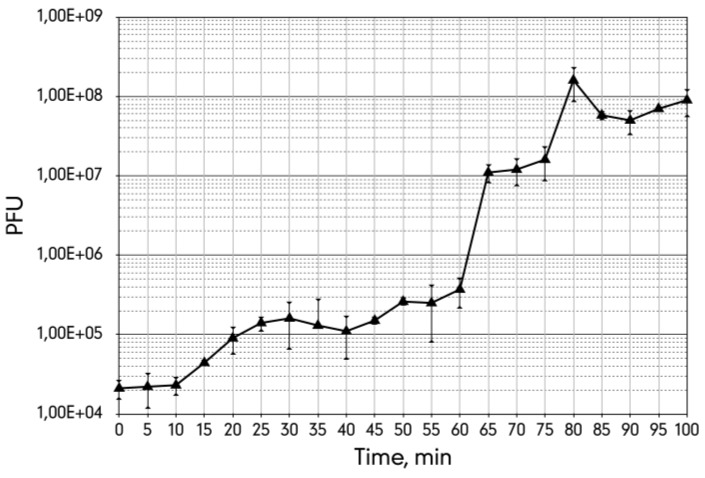
The single burst experiment curve of the bacteriophage 9g grown on *E. coli* C600 host at 37 °C with agitation at 250 rpm.

The single burst experiment indicated that the latent period of the phage 9g infection is about 60 min with the burst size of approximately 400 phages per cell (Figure 3). However, the plateau after the first cycle is only 15 min long and indicates that the infection is significantly non-synchronous. Presumably a substantial fraction of the infected cells liberate phage much earlier (*ca.* a 4–5-fold increase in the total phage count is observed at 20–25 min post infection) and thus restart the life cycle before the bulk of the progeny of the first infection is produced.

### 3.4. The Metastable Pseudolysogenic Associations of Phage 9g and Host.

The genetic organization of the phage 9g genome (see below) made us suspect that this virus can either lysogenize or instead enter into an unusual pseudolysogenic state characterized by partial repression of closely located promoters that might allow infected cells to divide, producing either infected progeny or a mixture of infected and non-infected cells.

**Figure 4:**
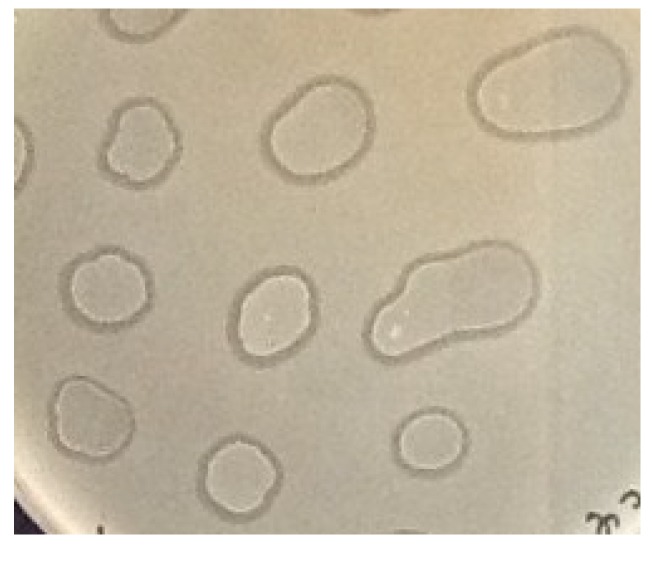
Test of phage 9g-*E. coli* C600 PAs (passage N° 15) for phage production on lawns of *E. coli* C600. 5 μL drops of the PA suspension in physiological saline were applied over lawns of *E. coli* C600. Lysis of the lawn around the drops indicates the presence of the active phage.

The plaques formed by 9g phage on *E. coli* C600 lawns as well as on the lawns of the *E. coli* 4sR and 4sAs strains are 2 mm in diameter, with clear centers and narrow turbid edges. Using toothipicks, we transferred material from the 20 plaques formed on *E. coli* C600 lawns, as well as 20 plaques formed on *E. coli* 4sAs, onto fresh LB plates. Weak growth of the culture was observed the next day. The repeated passages yielded, however, normally growing cultures. At every passage we transferred the cultures on both LB plates and on double layer plates with the freshly inoculated lawn of the original strain to visualize the presence of active phage particles. The ability to produce the phage was stably maintained in all of the cultures tested over at least 15 passages (Figure 4). We called these systems “pseudolysogenic associations” (PA) due to their apparent similarity to the characteristics of true lysogenic cultures.

Two PAs from each host strain were resuspended in physiological saline and plated at appropriate dilutions to form individual subclones. The subclones were tested for their ability to produce phage on the original strain’s lawn. All the subclones out of 100 tested for each host strain retained an ability to produce phage that was inherited over several subsequent passages. This result indicates a tight association of phage 9g and host cells. However, when the same procedure was repeated using instead of physiological saline the viricidal tea buffer that effectively inactivates free phage particles but not the bacterial cells, infected or non-infected (see *Materials and Methods*), all of the subclones produced were free of the phage. The absence of phage from the subclones was confirmed by PCR for gene 51 of phage 9g. Thus, we show that infected cells with internalized phage 9g DNA are not able to produce viable progeny and all the phage in our metastable phage-host associations therefore are maintained either in the form of free phages or instead in the form of lytically infected cells. Despite its ability to efficiently form into metastable associations with its hosts, phage 9g nonetheless can be classified as a true virulent phage.

### 3.5. Overall Genome Organization of Bacteriophage 9g

**Figure 5:**
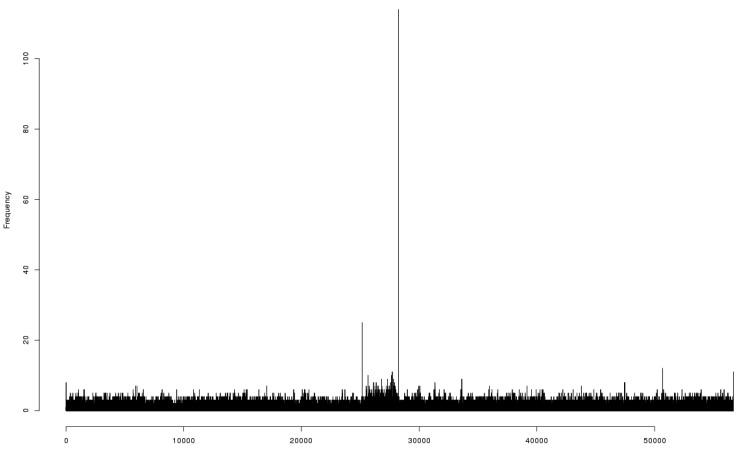
The diagram of the distribution of the individual sequencing reads starts and ends over the originally assembled sequence of the bacteriophage 9g genome.

DNA was extracted from bacteriophage 9g particles and submitted for high throughput sequencing using Ion Torrent^®^ technology (Life Technology). The resulting reads were successfully assembled into a single contig. Bacteriophage 9g’s unique genome sequence comprises 56,703 bp. Identification of the putative end position was based on mapping of the individual reads that start or end in each position of the generated sequence. The nucleotide position 28,243 was a clear hot point in the distribution of the individual reads starts/ends: 91 reads were initiated at this position (all in reverse orientation) and 23 reads ended at the position 28,243 (mean frequency of initiation and termination was *ca.* 2.6 reads per nucleotide position) (Figure 5).

The 85 reads that passed through this position were distributed almost evenly between the direct and reverse orientation. The existence of the reads that cross the putative physical end of the genome indicate the presence of the direct terminal repeat. However, the size of this repeat could not be evaluated from the coverage distribution since the half of the genome containing the early genes had *ca.* 1.5 more of coverage than another half, with the sharp break at the position 28,243. Taking together all this data, we conclude that the nucleotide position 28,243 represents a physical left end of the encapsidated phage 9g DNA with the packaging direction leftward. The genomic sequence was then linearized and reverse-complemented to make the genetic map collinear with the physical structure of the virion DNA.

**Figure 6:**
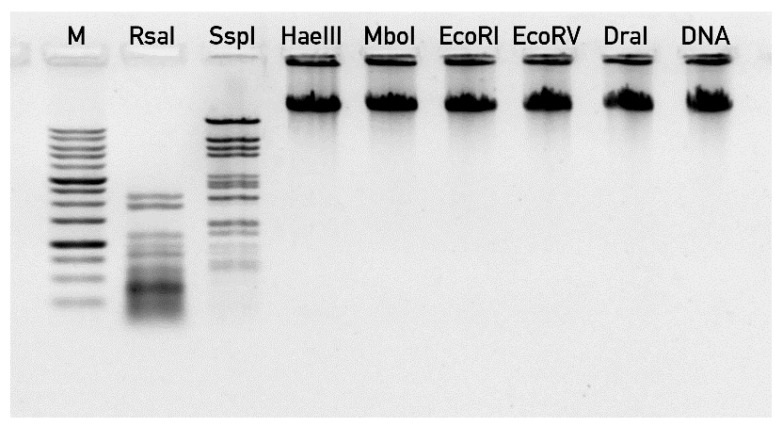
The digestion of bacteriophage 9g DNA by restriction endonucleases. “M” lane—1 kb DNA ladder (SibEnzym, Novosibirsk, Russia), “DNA” lane correspond to undigested 9g DNA.

Direct experimental validation of DNA end positions was hindered by the fact that phage 9g DNA was resistant to almost all the restriction endonucleases in our collection (including EcoRV, HaeIII, DraI, EcoRI, MboI), despite the presence of the corresponding recognition sites in the genome sequence. Only RsaI and SspI enzymes digested effectively this DNA (Figure 6), but they produced band patterns that were not suitable for prediction of DNA ends. This resistance of the phage DNA to multiple endonucleases suggests that one or more of the DNA bases are massively modified.

**Figure 7:**
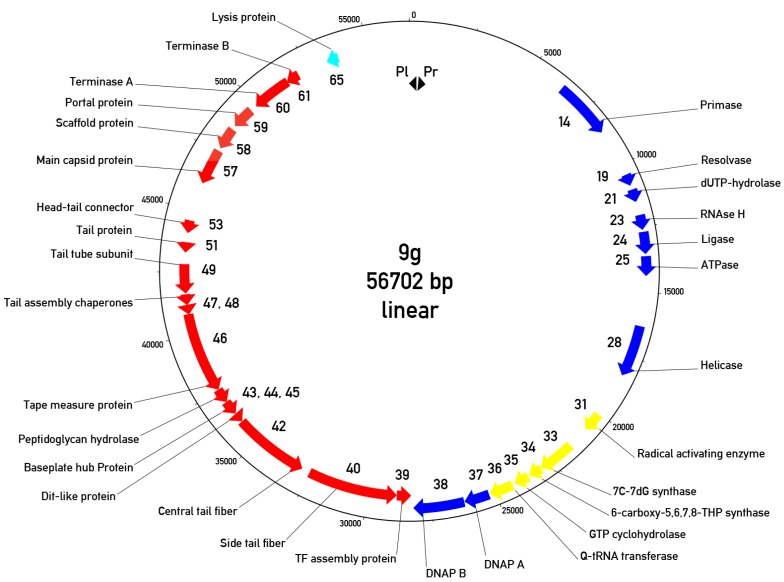
Circularized genomic map of bacteriophage 9g. The “0” is placed at the putative left end of the encapsidated virion DNA. Only the genes with predicted functions are indicated. Cyan arrow—Lysis protein, red arrows—Morphogenetic proteins, blue arrows—DNA metabolism proteins, yellow arrows—Queosine synthesis gene cluster. Pl and Pr—Left and right promoters.

Bioinformatic analysis of phage 9g genomic sequence identified 70 potential protein coding regions. Putative functions of at least 31 ORFs out of 70 predicted ORFs in the phage genome could be assigned using the BLAST search. The genomic map reveals two major transcription regions (Figure 7) that are almost identical in their lengths. 

The left region is directly (with respect to the map orientation, chosen so that is the same as the proposed DNA ejection direction) oriented and contains 37 genes responsible for the early (or early/middle) functions such as DNA synthesis, transcription regulation and nucleotide metabolism. The second region is transcribed in the opposite direction and encodes the late morphogenetic and lysis proteins. We predict two closely positioned promoters that are situated in a 473 bp non-translated region at the very left end of the genome. These promoters are oriented in opposite directions so that only the early region could be transcribed immediately after DNA entry into the host cell. However, the similar region is likely to be a part of the right terminal repeat so the switch between early and late transcription in phage 9g should be more sophisticated. In agreement with this prediction, we identified two putative transcription regulator proteins. One of them (g3) is located in the early region while the second (g70)—surprisingly—is found at the right end of the genome, presumably transcribed with the rest of the late genes.

It should be mentioned that once a concatemeric or circular phage 9g DNA replication intermediate is formed, the 603 bp intergenic region containing the divergently oriented promoters will be situated exactly between the early and late regions. Such an organization evokes the typical gene layout of the temperate phages, but in spite of the extensive search performed we found no detectable integrase-like genes. The presence of the phage encoded DNA polymerase also in not a typical trait of the temperate bacteriophages.

### 3.6. The Early or Early/Middle Functions

The left early or early/middle transcription region is 28 kbp long and encodes the set of DNA replication proteins: primase, dUTPase, resolvase, ATP-dependent DNA ligase, RNAaseH, two DNA polymerase subunits, putative DNA/RNA helicase and some other related functions. Protein gp3 is similar to the TetR family of transcription factors and may be involved in the early-late transcription switch. Some other proteins (gp15, gp17) also contain predicted DNA binding motifs and may be involved in transcription regulation.

Probably the most unusual feature of the 9g genome is the inclusion of a whole functional gene cluster encoding the full metabolic pathway of biosynthesis of a rare nucleoside, queuosine (7-deazapurine)—Genes 31–35 (organic radical activating enzyme, 7-cyano-7-deazaguanine synthase, 6-carboxy-5,6,7, 8-tetrahydropterin synthase, GTP cyclohydrolase) [32]. The phage also has a gene for the queuosine tRNA-ribosyltransferase (g 36). At the same time, no tRNAs genes were detected in the bacteriophage 9g genome.

### 3.7. The Morphogenetic Proteins and Other Late Functions

The late region has almost exactly the same length as the early region (28 kbp). Using pBLAST and PSI-BLAST searches, HHpred analysis and taking into consideration the established conserved gene order in the tail region in the majority of Siphoviruses [30], we were able to identify the main head-forming proteins (the portal, the main capsid protein, putative scaffolding and the putative head completion proteins; Figure 7) as well as small and large terminase subunits. The tail cluster includes identified tail tube protein, tape measure protein, distal tail proteins Dit and basal hub protein (BHP), responsible for the tail terminal conical structure formation [30] and the proteins responsible for the central and the side tail fibers, with the cognate chaperone for the latter one. Tail assembly chaperone G/T proteins that are encoded by nested genes with a programmed translational frameshift [33] were also detected. It is interesting to note that the ribosome slippage motif in phage 9g, 5’-CCCUUUUUUU (sequence confirmed by targeted Sanger sequencing), programs a -2 translational frameshift in contrast to a -1 frameshift occurring in the majority of the phages ([33] and [34]). 

Only one lysis-related protein (gp65) that is similar to holins, lysis endopeptidases or Rz proteins from many Enterobacteriaceae prophages was detected [35]. We were not able to distinguish between these possible functions of that gene. Despite the fact that we were not able to identify the complete lysis module, phage 9g induces effective lysis of the infected culture, suggesting that it may encode an unusual lysis mechanism. 

### 3.8. Relations of the Phage 9g to Other Bacteriophages

At the nucleotide level, the genome of bacteriophage 9g produces no significant matches to any GenBank entry. At the protein level the morphogenetic proteins of 9g are distantly related to phage phiJL001 [12]. The blastx search for the whole phiJL001 genome against predicted proteins of phage 9g identified reliably the relatedness of 10 genes and ORFs (listed in the order of phiJL001 genomic map): g39 (tail fiber assembly chaperon) g60 (large terminase subunit, g59 (putative portal protein), g58 (hypothetical proteins), gene 57 (main capsid protein) g55 (hypothetical protein), g55 (putative head to tail connector), g51 (tail protein), g49 (tail tube subunit) and g46 (tape measure protein). The level of identity within the aligned protein sequences was 24%–51% with the maximum value for the main capsid protein (MCP). In contrast to MCP the DNA polymerase sequences of these viruses do not match each other in the BLAST search (except for a 35 amino acid stretch in DNA polymerase subunit 1). The overall gene order as it can be judged from identified homologous protein is conserved in the late regions of these two phages. Bacteriophage phiJL001 was classified as a YuA-like virus [12], see also [9,36]. The terminase large subunit and the tail proteins mentioned above are also related between phages YuA, M6 and 9g and their order appears to be conserved. At the same time MCPs and portal proteins of the phages 9g and phiJL001 do not match respective proteins in phages YuA and M6. We conclude therefore that the genomes of the bacteriophages phiJL001 and 9g are too distantly related to belong to the same genus-level group. Moreover, both of them are likely to be a mosaic of modules of different origin.

## 4. Discussion

Bacteriophage 9g, being a relatively small siphovirus, nevertheless demonstrates a large set of unusual biological properties. The presence of modified bases in DNA is a more common feature of Myoviruses with large genomes, such as T-even phages, rather than of a member of the Siphoviridae family. It should be mentioned that phage phiJL001 [12], which is distantly related to 9g, contains a gene for a phage T6 Beta-glucosyl-HMC-alpha-glucosyl-transferase-like protein (JL001p22), but no enzymes that could be involved in the synthesis of unusual bases were detected, and phiJL001 DNA was normally digested by restriction enzymes. 

The presence of the queuosine synthesis cluster is also unusual, but not a unique property of phage 9g. Similar gene clusters were found in *Streptococcus* phage Dp-1 and in some other bacterial viruses [37]. Queuosine and its derivatives occur exclusively at position 34 (the wobble position) in the anticodons of tRNAs coding for the amino acids histidine, aspartate and tyrosine. Each of these tRNAs possess the anticodon sequence GUN (positions 34–36), where N can be any nucleotide, and queuosine improves the accuracy of translation. The tRNAs of the “Q-family” contain guanine in the first position of the anticodon, which is post-transcriptionally modified with an irreversible insertion during maturation. Queuosine cannot be synthesized by eukaryotes, but can be salvaged by them from diets or instead from their intestinal microorganisms, including enterobacteria (NCBI Biosystems BSID: 545485).

The nature of the selective advantage for a phage that might result from queuosine synthesis is not known. It was proposed that these genes are involved in the modification of the Q-containing tRNAs pool in the infected cell or even make up part of a sophisticated mechanism regulating the production of phage structural proteins at the level of translation [37]. However, all of these proposed functions remain speculative. The use of queuosine synthesis machinery to produce the modified base that is incorporated into the viral genomic DNA has not been, to our knowledge, reported previously. The exact nature of the modified base in phage 9g DNA has yet to be identified, but its genome contains no other candidates for unusual DNA base synthesis enzymes besides the queosine cluster mentioned above. It is interesting that the phage DNA is resistant to both HaeIII (GGCC) and DraI (TTTAAA) enzymes. This feature suggests that massive DNA modification is present.

The ability to form stable associations with their host is also an unusual aspect of bacteriophage 9g biology. The associations were formed in 100% of the cultures and maintained phage production in at least 15 passages (some of these cultures are still maintained in our lab, indicating that the stability of the phage-host associations is probably unlimited). This feature is strikingly different from the behavior of other horse-derived phage-host systems that we have previously tested in the same way. For example, out of 54 associations of the bacteriophage G7C, only five produced phage that were active on the original strain at passage number 8 and none after 13 passages; other phages do not form such metastable associations at all ([38], our unpublished data). We use the term pseudolysogenic associations (PA) for these cultures to highlight the phenotypical similarity to the true lysogenic cultures. It has to be mentioned that the term “pseudolysogeny” is used in the literature in multiple senses [39] that are not restricted to situation where a cell infected by a virulent phage proceeds through some limited number of the divisions. Moreover, this scenario has been convincingly demonstrated only in very few phage-host systems. The original understanding of the “pseudolysogeny” by G. Stent and d’Hérelle [39,40] and references therein is closer to the phenomenon observed by us in the *E. coli*-phage 9g systems. We believe therefore that the term “pseudolysogenic association” is an adequate description of the phenomenon observed. It is currently not clear what mechanisms stabilize the co-existence of high concentrations of phage 9g particles and succeptible host cells in such a dense community as an agar culture. The ability of PAs to proliferate *via* dispersal of the small fragments (possibly single cells), as it was demonstrated in the experiment with plating of the diluted suspensions of PA without viricide treatment, may be of ecological importance. This mode of stable growth of the host and phage in the form of colonies or microcolonies without extinction of the host population or replacement of the total host population by a phage resistant mutants may explain the stable maintenance of some virulent phages in the horse gut ecosystem (see refs [1,4]) despite the occurring drop of the overall concentration of the phage and suitable host below the expected phage replication threshold. It has to be noted that due to very high intraspecies diversity of the individual *E. coli* populations in horses, only a small fraction of *E. coli* cells can be infected by any given coliphage strain present in this system [1,8].

Summarizing all the data, we conclude that bacteriophage 9g by its biological properties and genomic sequence is clearly distinct from all previously described phages, and could be considered as a representative of a novel genus.
